# Case Report: Suppurative Labyrinthitis Induced by Chronic Suppurative Otitis Media

**DOI:** 10.3389/fneur.2022.892045

**Published:** 2022-06-09

**Authors:** Qianwen Xiao, Yuzhong Zhang, Jingrong Lv, Jun Yang, Qing Zhang

**Affiliations:** ^1^Department of Otorhinolaryngology-Head and Neck Surgery, Xinhua Hospital, Shanghai Jiao Tong University School of Medicine, Shanghai, China; ^2^Ear Institute, Shanghai Jiao Tong University School of Medicine, Shanghai, China; ^3^Shanghai Key Laboratory of Translational Medicine on Ear and Nose Diseases, Shanghai, China

**Keywords:** suppurative labyrinthitis, hearing loss, vestibular disorder, semicircular canals, cochlea, fistulae

## Abstract

A discussion on suppurative labyrinthitis associated with chronic suppurative otitis media (CSOM) may seem to be an outdated issue due to the advent of antibiotics in the last century. In previous literature, limited cases of suppurative labyrinthitis have been reported. This case, therefore, is an excellent and rare opportunity to study its clinical symptoms, diagnoses, and treatments. This report described the case of a 39-year-old female with a history of CSOM for 20 years, and she often presented with otorrhea, vestibular disorder, and hearing impairment. CT of the temporal bone revealed fistulae in both the basal turn of the cochlea and the horizontal semicircular canal. Combined with the otolaryngology examination results, suppurative labyrinthitis was considered. During a three-month follow-up, her symptoms were improved significantly after surgery. In conclusion, suppurative labyrinthitis must not be overlooked and neglected; early diagnosis and treatments are of vital importance.

## Introduction

Suppurative labyrinthitis is an inflammatory process involving bacterial invasion of the cochlea and vestibule (1). Bacteria can invade into the inner ear through oval window, round window, or pathologic fistula of the labyrinth leading to necrosis of membranous labyrinth, endolymphatic hydrops and so on. Symptoms of the disease are characterized by vertigo, tinnitus, nystagmus, and hearing impairment in the presence of a middle ear infection (2). Suppurative labyrinthitis, as one of the complications of chronic suppurative otitis media (CSOM), has been greatly reduced because of the widespread use of antibiotics (3, 4). However, this kind of disease still occur, especially in less favored communities in developing and developed countries, with significant morbidity (1, 4). Previous study had indicated suppurative labyrinthitis was identified as the most disabling among complications induced by CSOM, in that, affected individuals might experience complete hearing loss, seriously affecting their quality of life (5). Therefore, this disease should be valued. We present the case of a female patient with suppurative labyrinthitis aimed to gain a better understanding of its clinical symptoms, diagnoses, and therapies.

## Case Description

A generally healthy 39-year-old female with a history of CSOM for 20 years was seen at the otorhinolaryngology department of a local hospital. She presented with a 2-month history of vertigo, tinnitus, hearing impairment, and perforation in her left tympanic membrane (TM) with foul-smelling purulent drainage. Each episode of vertigo lasted several seconds and worsened with activity and eased with inactivity. In severe cases of the vertigo, she had sometimes fallen to the floor, making it clear that the condition affected the quality of life of the patient. The local otolaryngologist prescribed ofloxacin ear drops, betahistine mesylate, gingko biloba extract, etc. The symptoms were relieved after treatments on nine consecutive days. However, these drugs treated the symptom, not the underlying problem. The perforation, drainage, and vertigo reoccurred once the treatments were stopped. Hence, those resolutions are temporary and it is necessary for the patient to search for a more effective therapy to treat the disease.

For further treatment, the patient was admitted to our hospital in February 2021. She underwent physical examinations and a detailed audiovestibular evaluation including pure tone audiometry (PTA), acoustic immittance testing, oscular (oVEMP) and cervical vestibular-evoked myogenic potentials (cVEMP), and head-impulse paradigm (HIMP). PTA was performed to evaluate the level of hearing loss. Acoustic immittance testing was performed to evaluate sound transmission function in the middle ear. The oVEMP was used to evaluate the functions of utricular and superior vestibular pathways, and the cVEMP was performed to evaluate the saccular and inferior vestibular pathway functions (6). For HIMP, it was performed to evaluate the functions of three semicircular canals (7). She also had CT of the temporal bone and MRI of the inner ear to find lesion sites intuitively.

**Figure 1:**
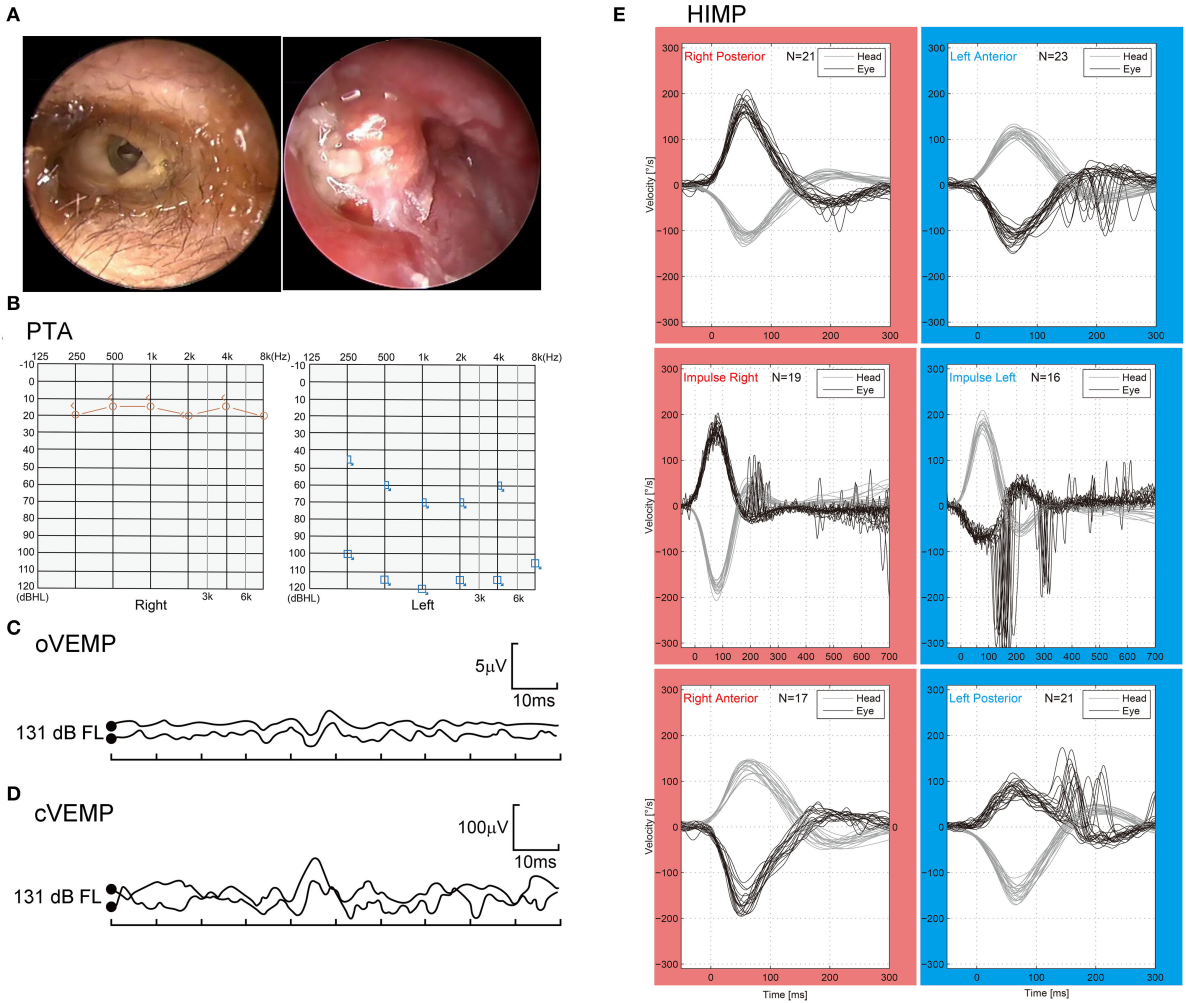
**(A)** Oto-endoscope showed perforation in the left tympanic membrane with formation of granulation tissue and cholesteatoma. **(B)** There was no response to air and bone conductions in the left ear. PTA, pure tone audiometry. **(C,D)** The VEMPs could not be elicited in the left ear. VEMP, vestibular evoked myogenic potentials; oVEMP: ocular VEMP; cVEMP: cervical VEMP. **(E)** The HIMP revealed reduced VOR gains of the lateral, superior, and posterior semicircular canals in the left ear. HIMP, head-impulse paradigm; VOR, vestibulo-ocular reflex.

**Figure 2:**
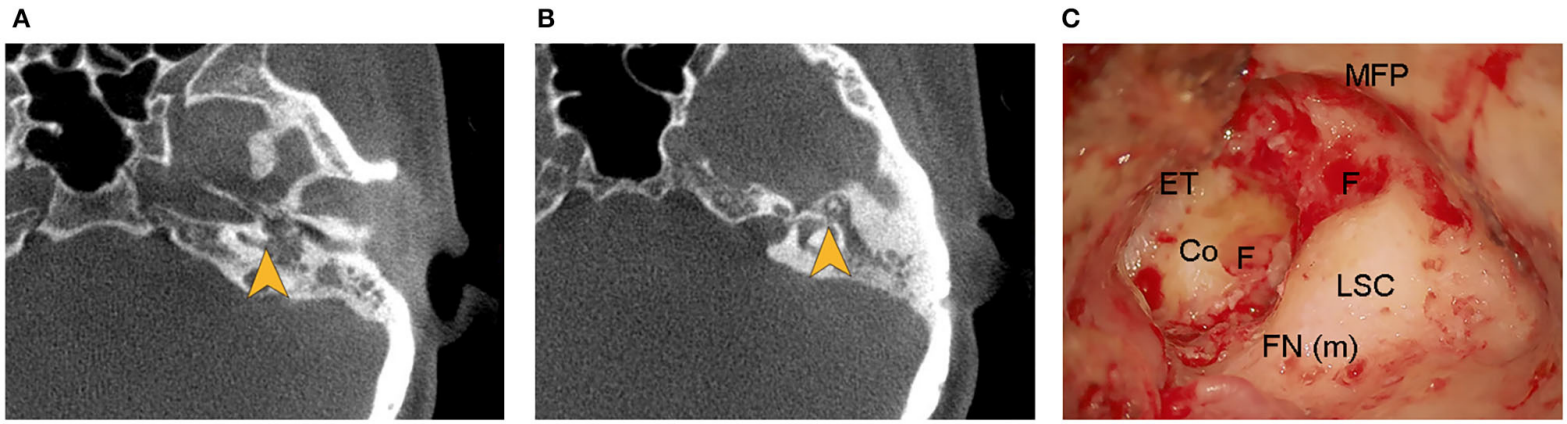
**(A,B)** Computed tomographic images of the temporal bone showed fistulae (yellow arrows) in both the basal turn of the cochlea and the horizontal semicircular canal. **(C)** Fistulae in both the basal turn of the cochlea and the horizontal semicircular canal were evident during the operation. Co, cochlea; ET, epitympanum; F, fistula; FN (m), mastoid segment of facial nerve; LSC, lateral semicircular canal; MFP, middle fossa plate.

On her physical examinations, Romberg's test was positive since the patient was unable to maintain an upright stance and her body leaned to the left side with eyes closed. Hennebert's sign was positive for the elicitation of pressure-induced nystagmus. The patient did not exhibit eye movements induced by sound, indicating the Tullio's phenomenon was negative. Oto-endoscope revealed an intact and clear right TM, and bloody and purulent secretions filling the left external auditory canal. Evacuation of the purulence showed perforation in her left TM with formation of granulation tissue and cholesteatoma (Figure 1A). Tympanometry examination showed a “B” type flat curve, indicating there was perforation in the tympanic membrane or blockage of the auditory canal (Supplementary Figure 1, Supplementary Video 1). The PTA illustrated there were no response to air and bone conductions in the left ear, but normal hearing in the right ear (Figure 1B). The patient showed absence of both oVEMP and cVEMP on the left side in bone-conducted vibration mode, with 500 Hz tone-burst stimulation delivered at 131 dB FL (Figures 1C,D). The VEMPs results elicited by air-conducted sound (ACS-VEMPs) and galvanic vestibular stimulation (GVS-VEMPs) are shown in Supplementary Figure 2. The HIMP displayed reduced vestibulo-ocular reflex (VOR) gains of the lateral, superior, and posterior semicircular canals during head impulses to the left side (Figure 1E). The CT scans of the temporal bone detected fistulae in both the basal turn of the cochlea and the horizontal semicircular canal (Figures 2A,B). Our MRI showed the vestibule and the cochlea were presented as nearly isointense on T1-weighted imaging (Supplementary Figures 3A,B). After contrast administration, the portion of lesion was slightly enhanced (Supplementary Figures 3C,D). Eventually, a clear diagnosis of suppurative labyrinthitis was made after combining with speciality test results.

**Figure 3:**
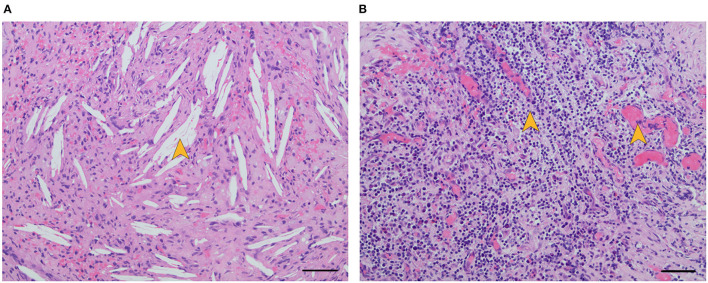
H&E staining of biopsy specimen showed cholesterol clefts [**(A)**, yellow arrow] and inflammatory granulation tissue formation [**(B)**, yellow arrows] around fistulae. Scale bar, 50μm (× 20).

The patient underwent tympanoplasty after significant reduction of otorrhea. Intraoperative exploration revealed the entrance of the tympanum was obstructed by large amounts of cholesteatoma and granulation tissue, and there was a large amount of granulation tissue with cholesteatoma epithelium in the posterior tympanum. The surgical procedure was complicated by the intraoperative finding of fistulae in both the basal turn of the cochlea and the horizontal semicircular canal during surgery (Figure 2C). Interestingly, we found the granulation tissue gushed out of the horizontal semicircular fistula when the cochlear fistula was pushed, indicating formation of granulation tissue in the labyrinth (Supplementary Video 2). The horizontal semicircular canal was packed with temporalis myofascial flap after clearing up granulation tissue and necrotic tissue. H&E staining of the biopsy specimen showed cholesterol clefts and inflammatory granulation tissue formation around the fistulae (Figure 3). The diagnosis of suppurative labyrinthitis was made again. The patient was discharged when antibiotic therapy was completed for 1 week after surgery. We provided important issues regarding maintaining a healthy lifestyle, including keeping the ears dry, avoiding scratching the ears, and persisting in vestibular rehabilitation training. During 3 months of follow-up, the patient was released from vertigo and otorrhea, and she expressed that surgery was a right choice. However, her hearing loss did not improve.

## Discussion

During the last 20th century, the introduction of antibiotics and immunizations led to a notable reduction in morbidity from complications related to otitis media (8). Intratemporal complications associated with otitis media mainly include labyrinthine fistula, mastoiditis, peripheral facial palsy, and labyrinthitis. Labyrinthitis is the least frequent complication with an annual incidence rate of 0.1% (4). Labyrinthitis usually consists of circumscribed labyrinthitis, serous labyrinthitis, and suppurative labyrinthitis. The identification of suppurative labyrinthitis is usually apparent through the observation of typical symptoms, which include hypacusis, tinnitus, vertigo, and moderate to severe mixed hearing loss (2, 3). Vertigo and disequilibrium are usually the presenting symptoms and can cause grave mortality. Vestibular neuritis and sudden deafness may also present with severe impairment of vestibular function, indicating that the diagnosis of suppurative labyrinthitis is not to be made based only on clinical symptoms (1, 9, 10).

Imaging tests are important tools to better understand pathological features and play an important role in diagnosis. Previous research has shown gadolinium in MRI is essential to detect inner ear inflammatory lesions and the temporal bone CT scans can identify lesion extent and location (11, 12). In our case, we found fistulae in both the basal turn of the cochlea and the horizontal semicircular canal through the temporal bone CT scans. This provides an important basis for us to quickly find the lesion site during the operation. We did find fistulae in both the basal turn of the cochlea and the horizontal semicircular canal during the surgery. H&E staining results of biopsy specimen showed the fistulae were filled with inflammatory granulation tissue and cholesterol clefts, indicating the patient is in the fibrosis stage of suppurative labyrinthitis (13). It is assumed that close contact of the infected granulation tissue and the underlying perilymph play a crucial role in the occurrence of physiopathological process (14, 15). However, there is no concrete and direct evidence of inflammation occurring during the period of suppurative labyrinthitis, and the studies of inflammatory response kinetics within the human inner ear are still limited (11). If we better understand the inflammatory process and its course, we can provide personalized treatments through identifying the type and the location of lesions. On 3 months of follow-up, our patient was released from vertigo after the surgery. We assumed that her vestibular symptoms were ameliorated through the process of vestibular functional compensation. Indeed, there is a spontaneous functional recovery because of neuronal and behavioral plasticity. For this reason, the intrinsic plasticity of the nervous system to reorganize may overcome the damages of the peripheral vestibular system, leading to a mitigation of vestibular symptoms (16). However, we found there was no improvement in hearing after surgery, suggesting once the inflammatory process developed into the inner ear, it resulted into an inevitable cell damage process (3). Thus, the hearing loss was already established when the patient was diagnosed with suppurative labyrinthitis. Regrettably, the patient missed the best time for diagnosis and treatments.

Early diagnosis and treatments of suppurative labyrinthitis are crucial to minimize the morbidity and mortality, and the doctor should be suspicious of this kind of disease when there are typical symptoms at present (15, 17). The decrease in morbidity and mortality may be attributable to advances in critical care, further studies on the mechanisms of inflammation, improvements in surgical techniques, close operation among clinics, and early referral of patients to otolaryngology.

## Data Availability Statement

The original contributions presented in the study are included in the article/Supplementary Material, further inquiries can be directed to the corresponding author.

## Ethics Statement

The studies involving human participants were reviewed and approved by Shanghai Jiaotong University. Written informed consent to participate in this study was provided by the participants' legal guardian/next of kin. The patients/participants provided their written informed consent to participate in this study. Written informed consent was obtained from the individual(s) for the publication of any potentially identifiable images or data included in this article.

## Author Contributions

QZ and JY were the surgeon in the case. QX and YZ contributed to the data collection and writing. QZ, JL, and JY guided the completion of this article. All authors contributed to manuscript revision and approved the submitted version.

## Funding

This study was supported by the National Natural Science Foundation of China (Nos. 81970891, 81970876, and 82171137).

## Conflict of Interest

The authors declare that the research was conducted in the absence of any commercial or financial relationships that could be construed as a potential conflict of interest.

## Publisher's Note

All claims expressed in this article are solely those of the authors and do not necessarily represent those of their affiliated organizations, or those of the publisher, the editors and the reviewers. Any product that may be evaluated in this article, or claim that may be made by its manufacturer, is not guaranteed or endorsed by the publisher.

## References

[1] Kaya S Schachern PA Tsuprun V Paparella MM Cureoglu S. Deterioration of vestibular cells in Labyrinthitis. Ann Otol Rhinol Laryngol. (2017) 126:89–95. 10.1177/0003489416675356 27881797

[2] Taxak P Ram C. Labyrinthitis and Labyrinthitis Ossificans: a case report and review of the literature. J Radiol Case Rep. (2020) 14:1–6. 10.3941/jrcr.v14i5.3706 33082921 PMC7536013

[3] Maranhão AS Godofredo VR Penido Nde O. Suppurative labyrinthitis associated with otitis media: 26 years' experience. Braz J Otorhinolaryngol. (2016) 82:82–7. 10.1016/j.bjorl.2014.12.012 26718959 PMC9444629

[4] Maranhão AS Andrade JS Godofredo VR Matos RC Penido Nde O. Intratemporal complications of otitis media. Braz J Otorhinolaryngol. (2013) 79:141–9. 10.5935/1808-8694.20130026 23670317 PMC9443832

[5] Leskinen K Jero J. Acute complications of otitis media in adults. Clin Otolaryngol. (2005) 30:511–6. 10.1111/j.1749-4486.2005.01085.x 16402975

[6] Tanyeri O Akdogan MV Hizal E Büyüklü AF. Assessment of vestibular function in adults with prelingual hearing loss using c/oVEMP tests. J Int Adv Otol. (2020) 16:24–7. 10.5152/iao.2019.7280 32066551 PMC7224435

[7] Chawla A Abdurahiman R Chokkalingam V. The video head impulse test: our experience in 45 cases. Indian J Otolaryngol Head Neck Surg. (2018) 70:498–504. 10.1007/s12070-018-1487-0 30464905 PMC6224837

[8] Wanna GB Dharamsi LM Moss JR Bennett ML Thompson RC Haynes DS. Contemporary management of intracranial complications of otitis media. Otol Neurotol. (2010) 31:111–7. 10.1097/MAO.0b013e3181c2a0a8 19887978

[9] Jeong SH Kim HJ Kim JS. Vestibular neuritis. Semin Neurol. (2013) 33:185–94. 10.1055/s-0033-1354598 24057821

[10] Lee JY Lee YW Chang SO Kim MB. Vestibular function analysis of sudden sensorineural hearing loss with dizziness. J Vestib Res. (2020) 30:203–12. 10.3233/VES-200703 32623411

[11] Floc'h JL Tan W Telang RS Vlajkovic SM Nuttall A Rooney WD. Markers of cochlear inflammation using MRI. J Magn Reson Imaging. (2014) 39:150–61. 10.1002/jmri.24144 23589173 PMC3935384

[12] Al-Juboori A Al Hail AN. Gradenigo's Syndrome and Labyrinthitis: conservative versus surgical treatment. Case Rep Otolaryngol. (2018) 2018:6015385. 10.1155/2018/6015385 30155332 PMC6091283

[13] Paparella MM Sugiura S. The pathology of suppurative labyrinthitis. Ann Otol Rhinol Laryngol. (1967) 76:554–86. 10.1177/000348946707600303 4963157

[14] Dubey SP Larawin V. Complications of chronic suppurative otitis media and their management. Laryngoscope. (2007) 117:264–7. 10.1097/01.mlg.0000249728.48588.22 17277619

[15] Osma U Cureoglu S Hosoglu S. The complications of chronic otitis media: report of 93 cases. J Laryngol Otol. (2000) 114:97–100. 10.1258/0022215001905012 10748823

[16] Lacour M Helmchen C Vidal PP. Vestibular compensation: the neuro-otologist's best friend. J Neurol. (2016) 263 Suppl 1:S54–64. 10.1007/s00415-015-7903-4 27083885 PMC4833803

[17] Wu JF Jin Z Yang JM Liu YH Duan ML. Extracranial and intracranial complications of otitis media: 22-year clinical experience and analysis. Acta Otolaryngol. (2012) 132:261–5. 10.3109/00016489.2011.643239 22224578

