# Supplementary material for: Case Report: Suppurative Labyrinthitis Induced by Chronic Suppurative Otitis Media
Source: Front Neurol. 2022 Jun 9;13:892045. doi: 10.3389/fneur.2022.892045 (PMC9218268; doi:10.3389/fneur.2022.892045)
Supplement: Supplementary file 3 [file Data_Sheet_1.docx]

**Supplementary Figure 1**: Tympanometry examination showed a “B” type flat curve in the left ear.

**Supplementary Figure 2**: The ACS-VEMPs and GVS-VEMPs results of the patient. Abbreviations: ACS: air-conducted sound; GVS: galvanic vestibular stimulation; VEMPs: vestibular evoked myogenic potentials.

**Supplementary Figure 3:** MRI showed the vestibule and the cochlea were presented as nearly isointense on T1-weighted imaging (**A, B**). After contrast administration, the portion of lesion was slightly enhanced (**C, D**).
