# Supplementary figures and images for: Case Report: Suppurative Labyrinthitis Induced by Chronic Suppurative Otitis Media
Source: Front Neurol. 2022 Jun 9;13:892045. doi: 10.3389/fneur.2022.892045 (PMC9218268; doi:10.3389/fneur.2022.892045)

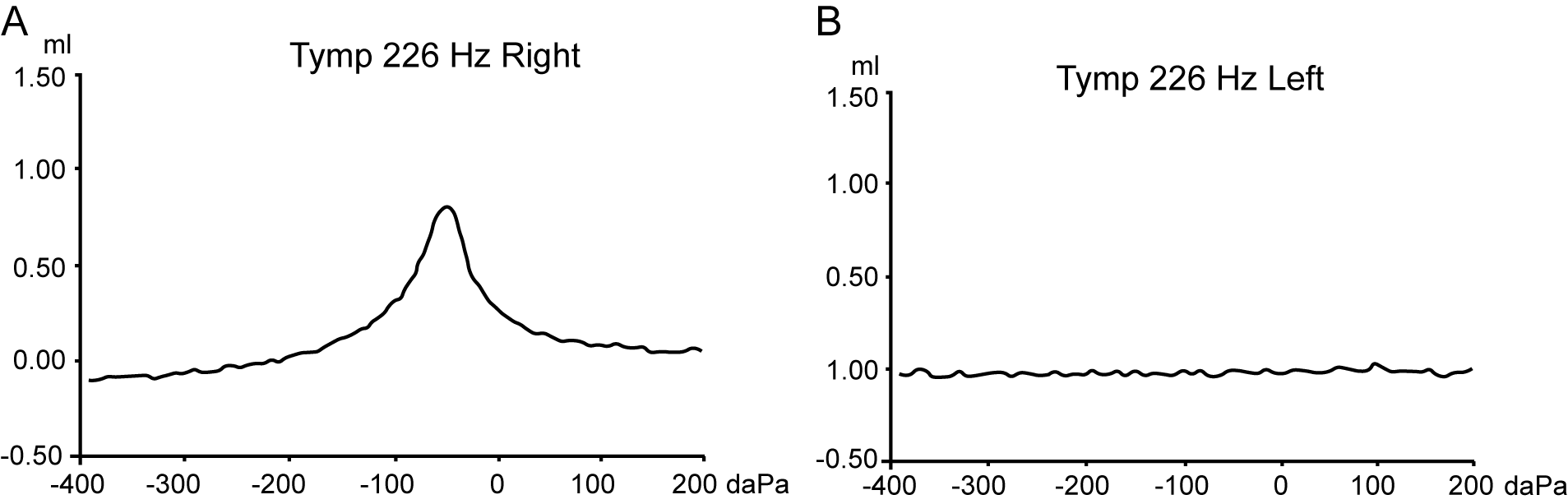

Supplement: Supplementary file 4 [file Image_1.TIF]

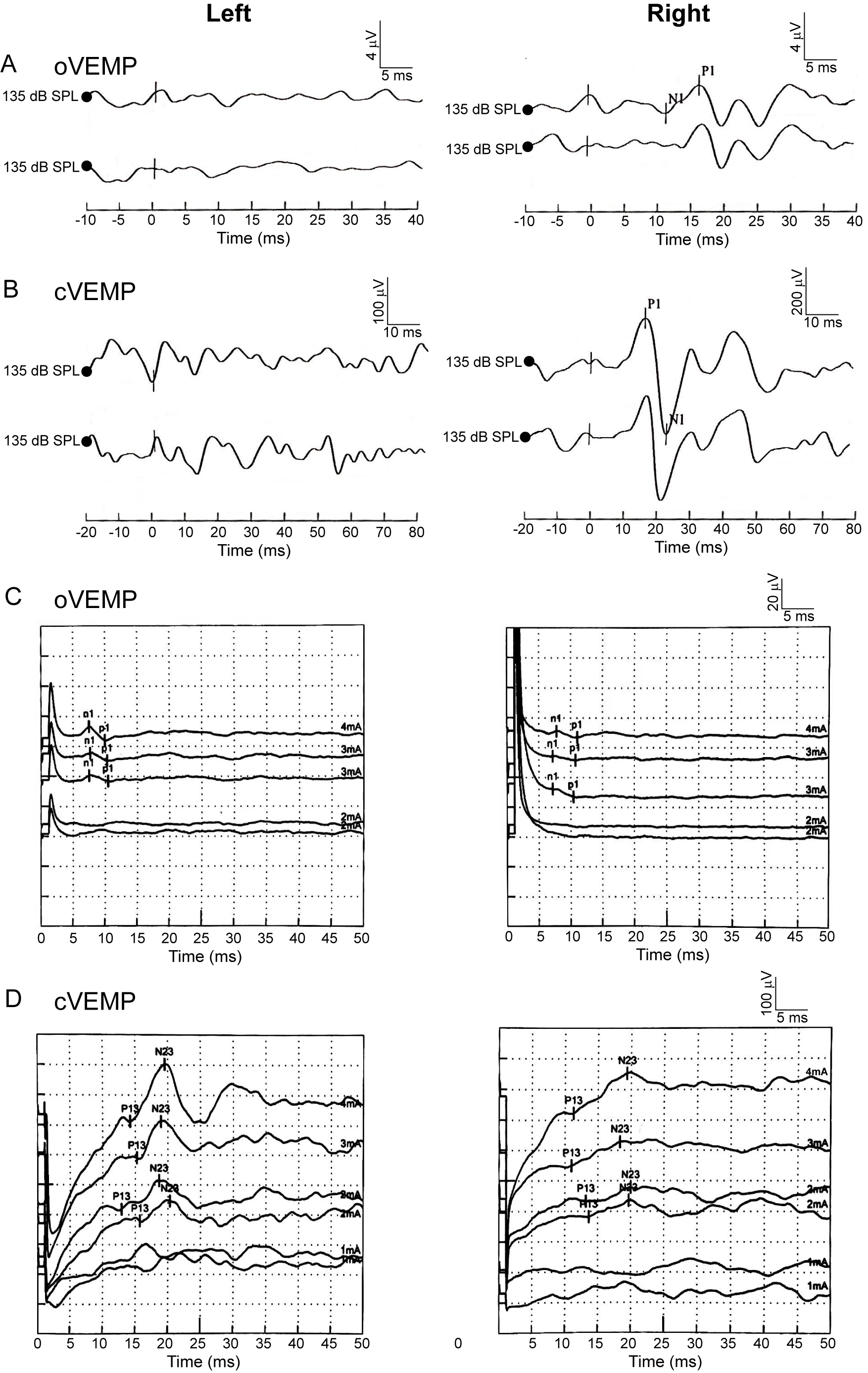

Supplement: Supplementary file 5 [file Image_2.TIF]

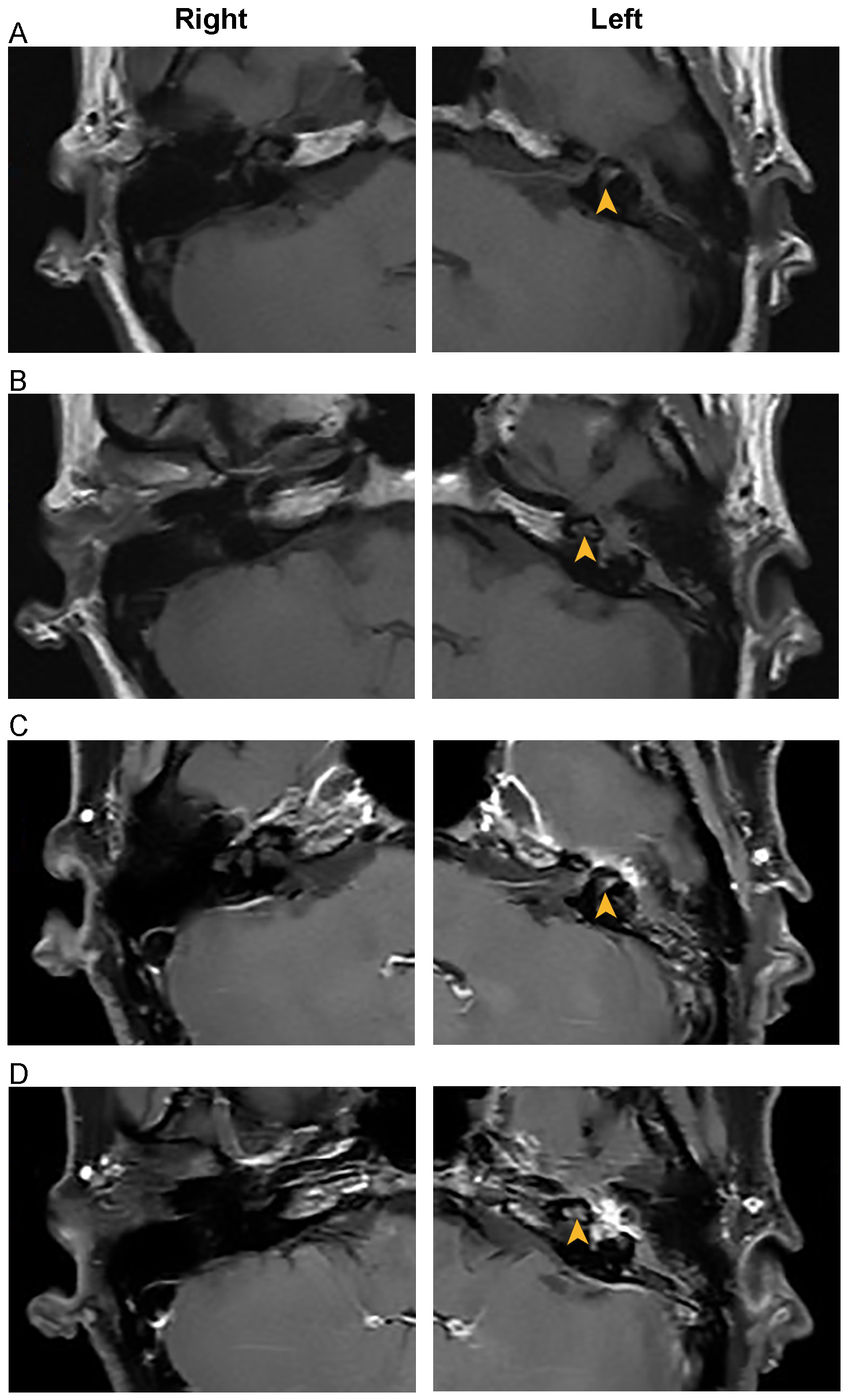

Supplement: Supplementary file 6 [file Image_3.TIF]
